# WNT signaling in microglia and the glioma microenvironment

**DOI:** 10.1186/2193-1801-4-S1-L50

**Published:** 2015-06-12

**Authors:** Gunnar Schulte

**Affiliations:** Karolinska Institutet, Sweden

**Keywords:** WNT, CNS inflammation, brain tumor

WNT signaling in microglia and the glioma microenvironment Gunnar Schulte WNT signaling is important during embryonic development and organogenesis having specific roles in the development of the CNS such as regulation of neural tube formation, axon guidance and CNS stem cell regulation. Our work has recently established a role of WNT signaling in the regulation of the brain’s macrophages, the microglia and thus WNTs emerge as novel regulators of CNS inflammatory responses. First of all, it appeared that b-catenin levels are elevated in microglia in Alzheimer disease (AD) brains as well as microglia cells in AD mouse models. Employing in vitro studies of primary mouse microglia isolated from newborn mouse pups indicated that both WNT-3A and WNT-5A induce diverse signaling routes in microglia leading to differential proinflammatory modulation of the cells. Interestingly, the net effect of WNT stimulation on the inflammatory potential of mouse microglia is context dependent. While WNTs increase inflammatory markers when giving to microglia alone, they are able to act in an anti-inflammatory manner when microglia are activated by prestimulation with lipopolysaccharides. Our findings thereby indicate that WNTs act on microglia as a homeostatic regulator, further underlined by yet unpublished data that suggest that WNT-5A is elevated in human glioma associated with a distinct inflammatory signature of the tumor as well as a substantial invasion of microglia.

